# Correction to: ‘Dolphin social phenotypes vary in response to food availability but not the North Atlantic Oscillation index’ (2023), by Fisher and Cheney

**DOI:** 10.1098/rspb.2024.2078

**Published:** 2025-05-08

**Authors:** David N. Fisher, Barbara J. Cheney

**Affiliations:** ^1^ School of Biological Sciences, University of Aberdeen, King’s College, Aberdeen AB24 3FX, UK; ^2^ School of Biological Sciences, University of Aberdeen, Lighthouse Field Station, George Street, Cromarty IV11 8YL, UK

**Keywords:** dolphin, environmental change, individual variation, plasticity, social network, Tursiops

*Proc. R. Soc. B* **290**, 20231187 (Published online 11 October 2023). (https://doi.org/10.1098/rspb.2023.1187)

We recently discovered a mistake in [1] with the use of one of the R functions for the social network analysis. One of the variables analysed was ‘closeness’, which we used to represent how well dolphins are connected to distant parts of the network (based on an individual’s direct and indirect connections). The function used to calculate closeness came from the *igraph* R package [2]. Since publication we have determined that this function treats the interaction strengths between individuals as distances or costs, where higher values mean more distant/less well connected. This interpretation of interaction strengths is opposite to how they are interpreted for most other social network metrics, where higher values indicate closer and more well-connected individuals, and opposite to how our interaction strengths are coded, where higher values indicate stronger associations.

The consequences are that the closeness values we analysed in the original version of the article are incorrect, and so the results and conclusions around closeness are erroneous.

We have re-calculated closeness using a different R package, tnet [3], which treats interaction strengths in the manner expected, i.e. higher values mean a stronger association, and re-ran all analyses involving closeness. The differences between the new (and correct) analyses and the previous (incorrect) set are shown in the table below. To summarize:

- There are now no relationships between salmon availability and closeness at the yearly or monthly scale (previously there was a negative relationship at the yearly and positive at the monthly).
- There is now no clear individual variation in slopes for closeness and the NAO index at the yearly or monthly scale.
- There is now a negative intercept–slope correlation for closeness and salmon availability at the yearly scale (previously positive).

Compared to the original version, the Correction has some changed text in the abstract, results, discussion, tables 1 and 2 and figures 2 and 3. The majority of our conclusions remain the same: dolphin social behaviour does not respond to the NAO index at either monthly or yearly scales, that dolphin social behaviour responds to monthly variation in food availability and different trait–environmental variable combinations show different degrees of among-individual variation in both means and slopes. What has changed is that dolphins no longer respond to yearly variation in any way (addressed in the discussion) and that only gregariousness responds to monthly food availability.

Additionally, while revising the correction, some very small further additional changes were made to the abstract and discussion. We have also updated the supporting data to reflect the revised dataset and included the datasets prior to filtering for the number of observations, allowing those interested to explore the effect this filtering has.

The following table summarizes the changes in results for closeness and the consequences these have had for the conclusions of the paper.

**Table:** 

time scale	environmental variable	variable	old result	new result	consequences
yearly	NAO	main effect	none	none	none
		interaction (sex)	none	none	none
		intercept–slope correlation and random slopes	negative and slopes present	no correlation or slopes	dolphins do not show individual variation in response to yearly NAO index values
	Salmon	main effect	negative	none	dolphins do not increase connectedness to wider parts of the network in years of low salmon availability, and so may switch among prey based on abundance rather than travel longer distances when salmon are scarce
		interaction (sex)	none	none	none
		intercept–slope correlation and random slopes	positive and slopes present	negative and slopes present	dolphins with below average mean closeness increase in closeness with increasing salmon abundance and those with above average mean closeness decrease closeness with increasing salmon; among-individual differences are greatest at low salmon abundance
monthly	NAO	main effect	none	none	none
		interaction (sex)	none	none	none
		intercept–slope correlation and random slopes	no correlation but clear individual variation in slopes	no correlation or slopes	dolphins do not show individual variation in response to monthly NAO index values
	Salmon	main effect	positive	none	dolphins do not increase connectedness to wider parts of the network in months of high salmon availability, and so response is simply in terms of increased gregariousness. (higher strength)
		interaction (sex)	none	none	none
		intercept–slope correlation and random slopes	none	none	none

**What follows now is a corrected version of the article.**

## 1. Introduction

Animals engage in social interactions with conspecifics, which are fundamental for determining health, access to resources and reproductive success [1]. Consequently, social interactions have a strong influence on ecological processes such as population dynamics and evolutionary processes such as the response to selection [2–4]. To maintain the best fit with their environment, animals may adjust their social behaviour as conditions change [5], for instance, being more gregarious when resources are plentiful but less tolerant of conspecifics when resources are scarce [6]. Animals may also change their behaviour through development and during senescence [7] and may non-adaptively adjust their behaviour due to the direct effects of the environment and other limitations [8]. Such plasticity is a hallmark of behavioural traits and gives behaviour an important role in how animals interact with their environment.

When trying to understand how animals may respond plastically to changing environments, most examine responses at the population level (e.g.[9]), presuming that any individual variation in response is absent or simply aggregates to give the population-level response. However, individuals may show variation in plasticity, and so each will respond differently to change ([10,11], sometimes referred to as ‘I × E’, i.e. individual-by-environment interactions [12,13]). For example, European field crickets (*Gryllus campestris*) become bolder and more active as they age, but individuals vary in the extent of this, with some not increasing or even decreasing [14]. The degree of plasticity animals show can be correlated with their mean behaviour (an ‘intercept–slope correlation’ (ISC)), which determines how the magnitude of among-individual differences varies across environments and indicates the extent to which plasticity is a separate trait in its own right [11,15]. Individuality in plasticity can influence biological processes such as population growth and adaptive change at a range of scales [16] (see [17] and accompanying papers), giving fundamentally different results when population-level only effects are assumed [15,18]. For example, Seebacher & Little [19] demonstrated that mosquitofish (*Gambusia holbrooki*) differ in how their performance changes with temperature, resulting in a switching of the rank of swimming speed of individuals between cool and warm temperatures. This changed which individuals might be predated, with a lower fraction of the population reaching the critical speed to avoid predation in cool temperatures [19]. Therefore, variation in plasticity will alter both the strength of selection and which genotypes produce phenotypes that are selected for, altering evolution trajectories. Additionally, the extent of among-individual variation in plasticity gives an upper limit to the heritability of plasticity, which indicates how rapidly plasticity itself can evolve [20–22]. Understanding how plasticity as a trait in its own right can evolve is key for understanding how animals will adapt to more variable climates [22,23].

Marine mammals are a key group to study individual variation in response to environmental change. They are typically long-lived, increasing the relative importance of plasticity versus adaptive evolution for coping with contemporary environmental change [24]. They are also exposed to a wide range of changing conditions during their lifetimes including climate, food availability and pollution, and their populations are often of conservation concern. All of these factors increase the need for us to understand how they respond to changes in their environment [25–32].

Here, we studied a population of bottlenose dolphins (*Tursiops truncatus*) in the North Sea for over 30 years, regularly recording their social associations. Previous work in this study population has demonstrated that critical group sizes increase in years of higher salmon abundance [33], and we extend this by examining multiple facets of individual-level social behaviour at both the monthly and yearly temporal scales. We achieved this by using social network analysis to quantify three different dimensions of individual social behaviour: an individual’s gregariousness (strength), how tightly its immediate social group interacts together (clustering coefficient) and how well connected an individual is to the entire population (closeness; each described in more detail below). We then used random regression models to quantify individual social phenotypes and determine how these social phenotypes depend on yearly and monthly variation in available proxies for climate (at a broad scale) and food availability (at a local scale). Our analyses also indicated whether bottlenose dolphins show individual changes in response to the environment or if population-level change was more prominent.

Specifically, we were interested in how social behaviour depends on current environmental conditions. Social behaviours are often highly dependent on both resource availability and spatial distribution [6], and current climatic conditions can impose energetic constraints on individuals [34,35] and impact their ability to move around their environment [36]. Bottlenose dolphin group sizes off the northwest coast of Spain showed a nonlinear relationship with the North Atlantic Oscillation (NAO) index [37], while abundances of Indo-Pacific bottlenose dolphins (*Tursiops aduncus*) are impacted by a combination of the El Niño Southern Oscillation and season [38]. In our study system, previous work indicated that the NAO index at a 2-year lag was associated with dolphin critical group size, but this appears to be entirely mediated through food availability [33], something we are testing for directly. As such, we did not consider lagged effects here. We summarized climate through the NAO index (see §2), where positive values in this region indicate warmer and wetter periods, which would make rougher sea conditions, potentially resulting in the dolphins travelling shorter distances. We, therefore, expect higher NAO values to lead to higher clustering coefficients and lower closeness but not to affect strength at monthly and yearly scales. We summarized resource availability through salmon abundances (see §2). We expect that higher salmon abundances allow dolphins to form larger groups (as found previously) and travel shorter distances to find sufficient food, leading to higher strengths, higher clustering coefficients and lower closeness at both temporal scales.

## 2. Material and methods

### (a) Study site and group data collection

This study used data from a bottlenose dolphin population on the east coast of Scotland (figure 1). The population of over 200 individuals [39] has been studied intensively as part of a long-term individual-based study [40–42]. We use data from boat-based photo-identification surveys carried out annually between 1990 and 2021, which regularly recorded dolphin groups within the Moray Firth Special Area of Conservation (SAC; 92/43/EEC), a core part of the population’s range which is over 50% of the population use each year [40]. All surveys were made from small (5–6 m) boats with outboard engines, carefully and slowly manoeuvring the boat around each group to obtain high-quality images of the left and right sides of as many dorsal fins as possible. Surveys initially followed a fixed survey route until 2001 when, as a result of changing dolphin distribution within the SAC, flexible survey routes were introduced to maximize sightings probability (more details in [43]). Data were available from a total of 690 surveys (between 9 and 35 surveys each year; average of 22), with the majority carried out between May to September.

**Figure 1.**
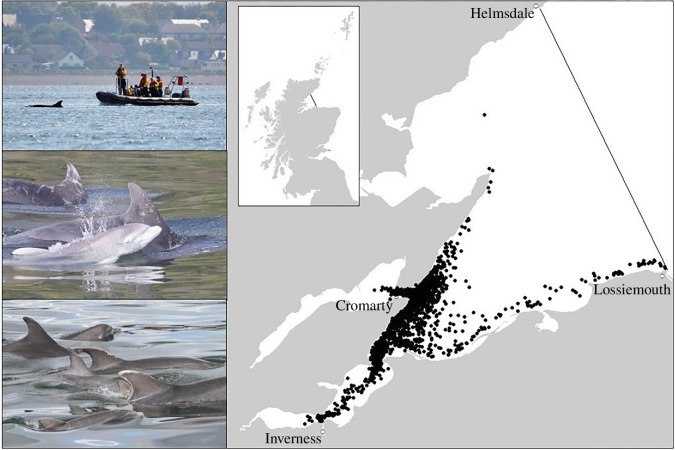
Images depicting a photo-identification survey (top left), a newborn calf with mother (middle left), unique markings used to identify individuals (bottom left) and location of encounters with bottlenose dolphins within the Moray Firth Special Area of Conservation from 1990 to 2021 (right).

During surveys, when we located a bottlenose dolphin group (one or more individuals in close proximity within 100 m, hereafter an ‘encounter’), we collected photo-identification data following a standardized protocol [43]. We identified individuals from high-quality photographs based on unique markings matched against a photo catalogue of previously identified individuals from the area [40,41,44]. On average, in a group, 84% are successfully photographed, a rate of identification well above the level at which social network metrics in incomplete networks are reliable [45]. All individual identifications from photographs were confirmed by at least two experienced researchers. For individuals first sighted as calves, we could determine their year of birth and so their age [46], but for individuals first sighted as juveniles or adults, their exact age is unknown. Sex was determined using genital photographs or if an adult was seen in repeat associations with a known calf [46].

### (b) Social network construction

Individuals sighted during the same encounter were assumed to be in the same, group, and therefore associating (known as the ‘gambit of the group’; [47]). Aggregating many of these records of groups allows one to infer which individuals are frequently associated and which individuals are infrequently or never associated. We removed observations of individuals younger than 3 years old (*n* = 2668 observations of 242 individuals), as these individuals are not likely to be independent of the mother, and so their social associations most likely represent her preferences. We then converted the records of encounters into group by individual matrices (indicating which individuals were seen together in each encounter) and then into weighted, undirected social networks using the R package *asnipe* [48]. Edge weights were set as the simple ratio index, where the number of times two individuals are seen together is divided by the total number of times they are seen, both together and apart [49]. This measure ranges from 0 (individuals never seen together) to 1 (individuals always seen together). We did this separately for each year, creating yearly social networks to assess how social phenotypes vary at this temporal scale in response to environmental conditions. To assess how social phenotypes vary at the monthly scale in response to environmental conditions, we then reconstructed social networks per month and removed any months with fewer than 10 encounters (excluding 110 out of 215 months; as networks constructed using fewer than 10 observations can be biased [50]). Histograms of the frequency of the number of encounters per year and per month are shown in electronic supplementary material, figure S1.

For each individual present in the network, for each year and again for each month, we calculated three network metrics. First was ‘strength’, the sum of all an individual’s associations, which, as our associations are based on observations of co-occurrence in groups, is analogous to the typical size of groups an individual is in. Second was ‘weighted clustering coefficient’, the rate at which an individual associates with other individuals who also associate with each other [51]. This metric represents how tightly individuals interact in their immediate social environment (possibly analogous to ‘alliances’ between three or more individuals; see also [52,53]), at the expense of interacting with a wider range of individuals. Finally, we quantified ‘closeness’, the inverse of the mean of the path lengths between that individual and each other individual in the network, corrected for network size to allow comparisons among networks, which vary in the number of individuals [54]. Closeness represents the dolphin’s connectedness to the wider population and would be high if an individual linked two communities or moved between different areas, each containing more sedentary individuals.

We removed an individual’s scores for a given year if they had fewer than five observations in that year (removing 811 observations and leaving 874), as the social network position of those individuals would be highly uncertain. They would, however, still contribute to the social environments and, therefore, social network measures of individuals in that year who had five or more observations. We repeated this at the monthly level, removing individuals’ monthly scores when they had fewer than five observations that month (removing 3423 observations and leaving 320). Histograms of the frequency of the number of encounters per individual per year and per month are shown in electronic supplementary material, figure S2. We had initially performed the analysis with a threshold of three observations before switching to the higher threshold of five; see the electronic supplementary material, tables S13–S24, for the results with the lower threshold. The social network measures showed moderate correlations; Pearson correlations between individuals’ strength and clustering coefficient were 0.141 (yearly) and 0.346 (monthly); for strength and closeness, they were 0.679 (yearly) and −0.573 (monthly); for clustering coefficient and closeness, they were 0.208 (yearly) and 0.579 (monthly).

### (c) Environmental data

We used the NAO index in the same time period the grouping observations were made as a measure of climate. We used monthly and yearly measures of the NAO index between 1990 and 2021, downloaded from https://www.cpc.ncep.noaa.gov/products/precip/CWlink/pna/nao.shtml (electronic supplementary material, figure S3). This index indicates the atmospheric pressure difference between the low-pressure zone over Iceland and the high-pressure zone over the Azores [55,56]. This index has frequently been linked to the ecology of animal populations [57,58], for example, influencing the foraging behaviour of Cory’s shearwaters (*Calonectris borealis*) [59]. Climatic effects on cetaceans are typically thought to occur via changes in prey species [31,60]; for instance, Lusseau *et al*. found the NAO at a 2-year lag influenced critical group size in our study population through the lagged variable’s effect on food availability [33]. However, it is also possible that cetaceans respond directly to climate, sometimes at even faster rates than their prey species [37,38,61].

For our index of food availability, we followed Lusseau *et al*. [33], using data on monthly catches by fishing rods (as opposed to nets) from the wild of both one-season and multiple-season adult Atlantic salmon (*Salmo salar*) from the Alness, Beauly, Canon, Ness and Nairn rivers. These feed into the sea where observations of dolphin groups take place and, hence, are expected to be a good proxy for salmon availability in that area. Further, catches on rods are positively correlated among rivers and among months and with automatic counter data, indicating they are a good proxy for actual abundance [62,63]. Atlantic salmon are an important food source for this dolphin population [64,65], with dolphins forming larger groups when salmon are more abundant [33]. We downloaded monthly data from https://marine.gov.scot/data/marine-scotland-salmon-and-sea-trout-catches-salmon-district-shinyapp (using ‘rod data’ and summing retained and released fish for both MSW and 1SW) and summed monthly catches within a calendar year for yearly measures of fish abundance (electronic supplementary material, figure S4).

### (d) Data analysis

All analyses were performed in R v. 4.3.1 [66] using linear mixed-effect models in *glmmTMB* [67]. Using regression-based models as opposed to randomization-based tests has been recommended for analysing questions about node-level social network traits as it improves the ability to make inference while accounting equally well as node permutations for common types of data non-independence [68]. We fitted 12 models, with all combinations across the three social traits, the two environmental variables and the yearly and monthly time scales. Clustering coefficient cannot be calculated when an individual only associates with one other individual, and so the datasets for these models were slightly smaller than the dataset for strength and closeness (see below). We included individuals with unknown birth and death dates to maximize our sample size, and so neither age nor lifespan could be included as predictor variables. In all models, we included the fixed effect of sex (as males and females can differ in social behaviour [69,70], ranging behaviour and survival [71]) and either the NAO index or the count of caught salmon for that month or year. We did not include both the NAO index and salmon abundance in the same model as we encountered estimation problems with two random slopes (RSs). Including sex meant we excluded individuals of unknown sex (140 observations of 66 individuals for the yearly networks, 30 observations of 21 individuals for the monthly networks), but the interactions between individuals of known and unknown sex were still used to build the networks, and so associations with individuals of unknown sex still influenced the social network traits of males and females. We mean centred and scaled to unit variance the environmental variable [72] and included the interaction between it and sex to see if male and female social behaviour responded differently to environmental variation. Random effects were the random intercept for individual ID, the RS of individual ID with the environmental variable fitted as a fixed effect in the model (again mean centred and with a standard deviation of 1) and the correlation between these two terms. We also included a temporal autocorrelation term (ar1) among years to account for unmodelled environmental variation that changes slowly across years, which could influence social behaviour to make adjacent years more similar than non-adjacent years. Similarly, in the models for monthly variation, we included a random effect of the month alongside the yearly temporal autocorrelation term. We used a Gaussian error structure for all models. For strength, we used log link functions as the distributions were right skewed. For clustering coefficient, which is bounded between 0 and 1, we used a logit link function, which is preferable to an arcsine transformation when handling response variables bounded in this way [73]. For closeness, we used the default link function. We used the default optimizing algorithm for all models except for the yearly models for clustering coefficient, the model for strength in response to monthly variation in the NAO index, the model for clustering coefficient in response to monthly variation in salmon abundance and both models for monthly variation in closeness, where we used the ‘BFGS’ optimizer, as otherwise, the models did not converge [67].

We report the coefficients and standard errors for each fixed effect, along with *p*-values, to give an idea of the magnitude and uncertainty of each effect. We used the *p*-values from the Anova function of the *car* package [74], using a Chi-squared test with type III sum of squares. We describe these *p*-values in terms of ‘clarity’ rather than ‘significance’; see Dushoff *et al*. [75] for a discussion on this. To test whether individuals clearly differed in their response to the environmental variable, we first tested whether there was a correlation between an individual’s plasticity and its mean behaviour by refitting each model (12 in total) with the correlation between random intercepts and slopes suppressed to zero and conducted a likelihood ratio test between the full model and this reduced model with a single degree of freedom. If there was a clear difference between the models, we concluded that the correlation between intercepts and slopes was non-zero. If there was no clear difference between the models, we then tested the importance of the RSs by comparing the model with an ISC of zero to a model without the RSs (but still with the random intercepts) using a likelihood ratio test with a mix of zero and one degree of freedom (as is appropriate for testing the clarity of single variance components; [76]). If there was a clear difference between the models, we concluded that individuals differ in their response to the environmental variable. When there is variation in plasticity, the magnitude of among-individual differences varies across environments. We, therefore, calculated the marginal repeatability for each trait following Schielzeth & Nakagawa [77]. We did this for all trait–environmental variable–time scale combinations (12 in total), even when there was no evidence for RSs, to aid comparison among traits. Data and R code are available online [78].

## 3. Results

### (a) Change with environmental variables at a yearly scale

For the analysis of how dolphin social phenotypes change at a yearly scale in response to environmental variables, our dataset included 129 unique individuals. For strength and closeness, there were 874 measures, and for clustering coefficient, there were 873 measures. Strength and closeness had a mean of 6.78 (s.d. = 5.39) measures per individual, while clustering coefficient had a mean of 6.77 (s.d. = 5.39) measures per individual.

Dolphins’ strength and clustering coefficient were not affected by the NAO index (figure 2*a,b*) or salmon abundance (figure 2*d,e*) in both sexes, and the sexes did not differ in mean strength or clustering coefficient (table 1; full model results in electronic supplementary material, tables S1–S4). For the relationships between both the strength and clustering coefficient and the NAO index, there were no ISCs, and the RSs were not statistically clear (table 2). Individuals did differ in how their strengths changed with salmon abundance, with a clear negative ISC (table 2). There was no ISC and no RSs for the change of clustering coefficient with salmon abundance (table 2). The marginal repeatabilities for strength were low: 0.017 in the NAO index model and 0.018 in the salmon abundance model, suggesting the trait has limited repeatability. The marginal repeatabilities for clustering coefficient were higher: 0.172 and 0.178 for the NAO index and salmon abundance models, respectively.

**Figure 2.**
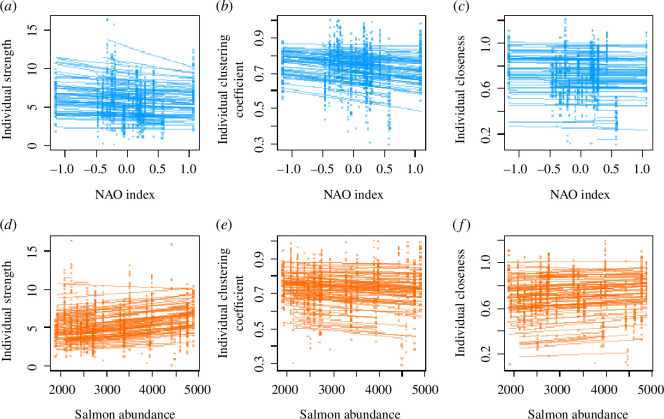
Plots of each of the three social network traits and yearly variation in the NAO index ((*a*) strength, (*b*) clustering coefficient and (*c*) closeness) and salmon abundances ((*d*) strength, (*e*) clustering coefficient and (*f*) closeness). For each individual dolphin, we have predicted its network trait on the observed scale based on the model results, using the ‘predict’ function in R with the individual’s sex, the range of NAO values or salmon counts that individual was exposed to, and picking a random year that individual experienced.

**Table 1.** Main effects (M) and interactions with sex (I) for the two environmental variables’ effects on the three social network traits at each of the monthly and yearly scales. Effects clearly different from zero (*p* < 0.05) are highlighted in bold, while effects with *p-*values between 0.07 and 0.05 are shown in italics.

			strength	clustering coefficient	closeness
NAO index	yearly	M	β = −0.035 ± 0.066, χ^2^ = 0.280, *p* = 0.597	β = −0.102 ± 0.081, χ^2^ = 1.595, *p* = 0.207	β = −0.001 ± 0.028, χ^2^ = 0.000, *p* = 0.985
		***I***	β = −0.002 ± 0.021, χ^2^ = 0.005, *p* = 0.942	β = 0.031 ± 0.035, χ^2^ = 0.793, p = 0.373	β = −0.004 ± 0.007, χ^2^ = 0.245, p = 0.621
	monthly	M	β = 0.053 ± 0.034, χ^2^ = 2.437, *p* = 0.119	*β = −0.102 ± 0.056, χ^2^ = 3.304, p = 0.069*	β = −0.007 ± 0.016, χ^2^ = 0177, *p* = 0.674
		***I***	β = 0.039 ± 0.037, χ^2^ = 1.105, p = 0.293	β = 0.057 ± 0.069, χ^2^ = 0.678, p = 0.410	β = 0.026 ± 0.018, χ^2^ = 2.073, p = 0.150
salmon	yearly	M	β = 0.108 ± 0.069, χ^2^ = 2.422, *p* = 0.120	β = −0.027 ± 0.091, χ^2^ = 1.595, *p* = 0.207	β = −0.021 ± 0.034, χ^2^ = 0.374, *p* = 0. 541
		***I***	β = −0.009 ± 0.026, χ^2^ = 0.126, p = 0.723	β = −0.070 ± 0.038, χ^2^ = 0.794, p = 0.373	β = 0.006 ± 0.017, χ^2^ = 0.720, p = 0.505
	monthly	M	**β = 0.137 ± 0.038, χ^2^ = 12.755, *p* < 0.001**	*β = 0.116 ± 0.062, χ^2^ = 3.569, p = 0.059*	β = 0.039 ± 0.029, χ^2^ = 1.804, *p* = 0.179
		***I***	β = −0.011 ± 0.045, χ^2^ = 0.065, p = 0.799	β = −0.105 ± 0.074, χ^2^ = 2.011, p = 0.156	β = −0.027 ± 0.022, χ^2^ = 1.510, p = 0.219

**Table 2.** Intercept–slope correlations and their statistical tests, and whether RSs were present or absent and their statistical tests (likelihood ratio tests in all cases, an asterisk is given for the test of the RSs if it was not performed as the ISC was first found to be clear). Clear negative ISCs are highlighted with bold text.

			strength	clustering coefficient	closeness
NAO index	yearly	ISC	−0.377 (χ_1_^2^ = 1597, *p* = 0.206)	0.996 (χ_1_^2^ = 1.274, *p* = 0. 259)	−0.003 (χ_1_^2^ = 0.000, *p* = 0.995)
		RS	absent (χ_0,1_^2^ = 1.688, *p* = 0.097)	absent (χ_0,1_^2^ = 0.023, *p* = 0.500)	absent (χ_0,1_^2^ = 0.117, *p* = 0.366)
	monthly	ISC	−0.976 (χ_1_^2^ = 0.868, *p* = 0.352)	0.075 (χ_1_^2^ = 0.000, *p* = 0.985)	−0.996 (χ_1_^2^ = 0.429, *p* = 0.513)
		RS	absent (χ_0,1_^2^ = 0.001, *p* = 0.486)	absent (χ_0,1_^2^ = 0.030, *p* = 0.431)	absent (χ_0,1_^2^ = 0.000, *p* = 0.500)
salmon	yearly	ISC	**−0.603 (χ_1_^2^ = 7.195, *p* = 0.007)**	0.998 (χ_1_^2^ = 2.660, *p* = 0.103)	**−0.826 (χ_1_^2^ = 9.379, *p* = 0.002)**
		RS	present*	absent (χ_0,1_^2^ = 0.000, *p* = 0.500)	present*
	monthly	ISC	**−0.769 (χ_1_^2^ = 6.191, *p* = 0.013)**	−0.967 (NA, model did not converge)	−0.999 (χ_1_^2^ = 2.221, *p* = 0.136)
		RS	present*	absent (χ_2_^2^ = 0.035, *p* = 0.983)	absent (χ_0,1_^2^ = 1.998, *p* = 0.079)

Closeness did not vary with NAO index or salmon abundance in either sex (figure 2*c,f* and table 1; full results in electronic supplementary material, tables S5 and S6). The sexes had similar closeness scores. Dolphins showed no mean–plasticity relationship for the NAO index, and there was no evidence for individual variation in slopes (table 2). In contrast, there was a negative ISC for closeness in response to salmon abundance (table 2), indicating that individuals with lower means increased their closeness as salmon abundance rose, while those with higher means decreased. The marginal repeatabilities were somewhat low for closeness: 0.112 in the NAO index model and 0.111 in the salmon abundance model.

For all social traits, there was substantial among-year variation (electronic supplementary material, tables S1–S6). Scores for all traits in consecutive years were positively correlated (year-to-year correlations: strength: *r*_NAO_ = 0.452, *r*_Salmon_ = 0.566; clustering coefficient: *r*_NAO_ = 0.590, *r*_Salmon_ = 0.657; and closeness: *r*_NAO_ = 0.656, *r*_Salmon_ = 0.663), showing that, as expected, adjacent years were more similar than non-adjacent years.

In summary, dolphin social behaviour did not respond to environmental variation at the yearly scale. For strength and closeness, individuals showed variation in plasticity in response to salmon abundance, which was negatively related to their mean behaviour but no such variation in response to the NAO index. For clustering coefficient, individuals showed no variation in individual plasticity, but they did show consistent differences between individuals across environments, which strength did not, while closeness showed a little repeatability.

### (b) Change with environmental variables at a monthly scale

We analysed how dolphin social phenotypes change in response to environmental variables at the monthly scale with a dataset of 88 unique individuals and 320 measures for all traits. Traits had a mean of 3.64 measures (s.d. = 2.64) each.

Strength increased with monthly salmon abundance for both sexes (figure 3*d* and table 1), and there was individual variation in plasticity and a negative ISC, with individuals with lower means increasing more than those with higher means (table 2). However, strength did not respond to the monthly NAO index (figure 3*a*), and there was no individual variation in plasticity due to the NAO (table 1; full model results in electronic supplementary material, tables S7–S12). As for the yearly models, the marginal repeatability of strength was low (0.003 in the NAO index model, 0.007 in the salmon abundance model).

**Figure 3.**
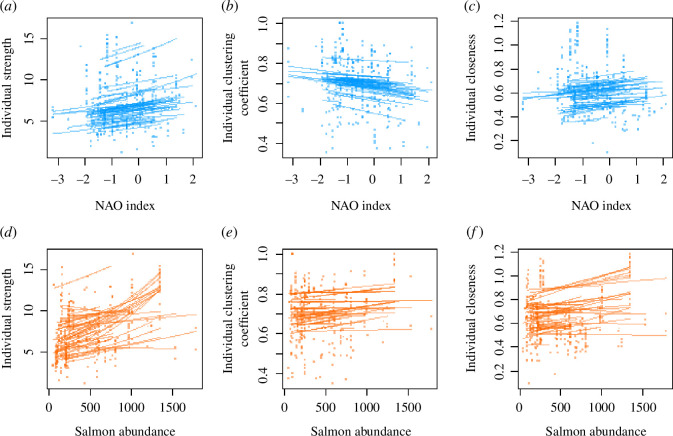
Plots of each of the three social network traits and monthly variation in the NAO index ((*a*) strength, (*b*) clustering coefficient and (*c*) closeness) and salmon abundances ((*d*) strength, (*e*) clustering coefficient and (*f*) closeness). For each individual dolphin, we have predicted its network trait on the observed scale based on the model results, using the ‘predict’ function in R with the individual’s sex, the range of NAO values or salmon counts that individual was exposed to, picking a random year that individual experienced, and the month to June (an arbitrary choice that was approximately in the middle of the calendar year).

Neither clustering coefficient nor closeness showed relationships with either the monthly NAO index (figure 3*b,c*) or the monthly salmon abundance (figure 3*e,f* and table 1). The sexes also did not differ in means for either trait. Individuals showed no mean–plasticity relationship and no individual variation in response to either variable for either trait (table 2; note that the model for clustering coefficient and monthly salmon would not converge with random intercepts and slopes but no correlation between them, so we compared the full model with the model without RSs using two degrees of freedom). Repeatabilities for both clustering coefficient and closeness were low, with values of 0.038 and 0.020 for clustering coefficient in the NAO index and salmon abundance models, respectively, and for closeness, 0.016 in the NAO index model and 0.006 in the salmon abundance model.

There was among-month and among-year variation in the monthly models, but social traits in consecutive years were either negatively or not correlated (year-to-year correlations: strength: *r*_NAO_ = −0.016, *r*_Salmon_ = 0.024; clustering coefficient: *r*_NAO_ = −0.307, *r*_Salmon_ = −0.399; and closeness: *r*_NAO_ = −0.183, *r*_Salmon_ = −0.166).

In summary, months with higher salmon numbers led to higher strength but not to a change in clustering coefficient or closeness. Meanwhile, the NAO index did not clearly affect any network trait. Individuals differed in how their strength changed in response to salmon abundance, with a negative mean–plasticity relationship. No other trait–environment combinations showed evidence of individual variation in plasticity. Clustering coefficient and closeness were very slightly repeatable across environments, but strength was not.

## 4. Discussion

We explored whether bottlenose dolphin social behaviour responded to environmental variation. Social behaviour responded to monthly variation in food availability, with overall gregariousness (strength) increasing in months of higher salmon abundances. Clustering of the local social environment (clustering coefficient) and connectedness to the wider network (closeness) showed no clear relationships with monthly salmon abundances, and none of the social traits responded to yearly salmon abundance or to climatic conditions at either time scale. In addition, we found that individuals showed consistent differences in mean clustering coefficient and closeness, especially at the yearly scale, but only closeness showed individual variation in plasticities. Strength showed some variation in individual plasticity but limited consistent differences in mean behaviour. For salmon abundance, this plasticity in strength and closeness was often but not always negatively associated with mean behaviour, causing among-individual differences to typically be greater for low values of salmon abundance.

Months of higher salmon abundance led to an increase in strength and positive but not clear trends for clustering coefficient and closeness (see figure 4 for a comparison of networks between months with low versus high salmon abundance). This indicates that dolphins were increasing all kinds of social associations (both existing local associations and connections more widely in the group) in response to increased immediate food availability. Similar results have been found in spotted hyenas (*Crocuta crocuta*), where seasons with high prey availability have denser social networks [79], and in Grant’s gazelles (*Nanger granti*), where increased rainfall (and so food availability) led to higher closeness scores [80]. It is presumed that an increase in social interactions at higher food availability is facilitated by a reduction in the intensity of resource competition (reviewed in [6], see also [81]). Therefore, in our study system, higher rates of social interaction may be beneficial for non-foraging reasons, such as mating. Both mating and calving seasons approximately align with the months of highest salmon abundance in the summer (electronic supplementary material, figure S4), supporting this suggestion. Additionally, dolphins could move into areas where food availability is especially high [42,82], causing more individuals to be seen together and, therefore, inferred social networks to be denser, even if actual rates of social interaction are not changing or only changing as a by-product. Finally, it is possible that months with fewer salmon also differ in an unidentified variable which causes dolphins to group less. However, it is unlikely that this unidentified factor is a predation threat that changes month to month, as predators are absent in this area [83]. Therefore, a change in social behaviour and/or movement related to the seasonal availability of salmon, perhaps influenced by reproductive behaviour, seems the most likely.

**Figure 4.**
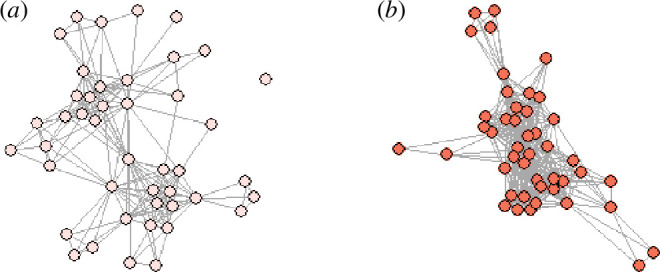
Plots of dolphin social networks in (*a*) a month of low salmon abundance (May 1990) and (*b*) a month of high salmon abundance (August 2007). Circles are individual dolphins, and grey lines indicate associations, i.e. those seen in the same group at least once in that month.

Interestingly, there was no change in any social trait in response to salmon abundance at the yearly scale. At the greater time scale, dolphins may not be responsive to salmon abundance as they can access alternative food sources. For instance, cod (*Gadus morhua*), saithe (pollock, *Pollachius virens*) and whiting (*Merlangius merlangus*) are all important food sources for dolphins in this population [65]. The presence of sufficient numbers of any of these when salmon abundances are low across a year and opportunistically feeding on the more abundant species would ameliorate the need for a shift in social behaviour. Common dolphins (*Delphinus delphis*) off the coast of northwest Spain, for example, show higher numbers of sardine (*Micromesistius poutassou*) and hake (*Merluccius merluccius*) in their diets when these species are more abundant, suggesting an opportunistic feeding strategy [84]. Whether such opportunistic feeding strategies are sufficient to maintain social structure or not, the fact that a change at one time scale can be absent at another is intriguing and should be kept in mind when attempting to generalize results from one time scale to another.

Despite the change in gregariousness in response to salmon abundance, we did not see any responses to climatic variation (the NAO index) on a yearly or monthly scale. The NAO index varied considerably at both scales, ranging from −1.15 to 1.08 at the yearly scale and −3.18 to 2.12 at the monthly scale (electronic supplementary material, figure S3); hence, a lack of necessary variability seems unlikely. Lusseau *et al*. [33] also observed no variation in group size in our study population in response to contemporary variation in the NAO index (they did see an effect at a 2-year lag, likely mediated by food availability). The robustness or inflexibility of social behaviour in response to variation in climatic conditions might indicate that the variation in the NAO index is inconsequential, and so they have no need to respond to it. Additionally or alternatively, dolphins may change other phenotypes, such as foraging behaviour or metabolism, to cope with this stressor [31], leaving social behaviour unchanged. Finally, local conditions might be more relevant to dolphin behaviour, as opposed to the regional conditions summarized by the NAO index. For example, the movements of bottlenose dolphins depend on tidal currents and fronts [85], and changes to these might be important for their social behaviour.

Alongside the plasticity at the population level in strength, this trait and closeness showed individual variation in plasticity in response to salmon abundance (although closeness only showed this at the yearly level). Therefore, even if the population as a whole showed no overall change for some trait–salmon abundance combinations, some individuals might still show an increase in their overall gregariousness and/or connectedness to wider parts of the network, while others a decrease. Variation in individual plasticity leads to environment-dependent repeatability (and possibly heritability), can dampen population responses to environmental variability and enhance population persistence [18]. For example, due to the negative ISC, individual strength shows the most among-individual variation at low salmon abundances (the approximate marginal repeatability of strength at the monthly scale two standard deviations below the mean salmon abundance was 0.124, compared with 0.02 at the mean). If strength is linked to foraging strategy, for instance, if individuals with more social connections have access to more information about prey availability [86], a wider range of social phenotypes could increase the possibility that at least some individuals are successful despite low food availability. Determining the genetic basis and importance of early life conditions for the development of these different responses [12] and how this variation impacts population dynamics [87] is key. Models projecting how these traits in dolphin populations will change in the future should account for both among-individual variation in mean behaviour and behavioural plasticity. Additionally, Kebke *et al*. [31] suggest that cetacean ranging and foraging behaviour may well be under selection for increased plasticity as environments change, and so estimating selection on both means and plasticities of behaviours is a logical and important next step.

In contrast, clustering coefficient showed no individual variation in plasticity at any time scale–environmental variable combination. Clustering coefficient at the yearly scale was the only trait with more than slight repeatability, indicating some individual consistency (see also [88] who found consistency over lifetime in the social behaviours of Indo-Pacific bottlenose dolphins, *T. aduncus*). Therefore, plasticity might be more limited with individuals keeping the same pattern of local connections across environmental conditions. As clustering coefficient depends on the frequency of connections among sets of three individuals, an individual’s trait value cannot change without impacting the trait value of others. This interdependence may then constrain the degree of plasticity possible at the individual level. There is no evidence for male alliances in this population [89], and so determining what these clusters of individuals represent and why they might be so stable would be useful.

In conclusion, we found that individual dolphin social behaviour responds to food availability, with overall gregariousness increasing in months of higher salmon abundance. In contrast, dolphin social behaviour showed no response to monthly or yearly variation in climatic conditions. Traits that showed higher repeatability tended to show limited individual variation in plasticity, although there was considerable variation in this trend. As such, whether individual heterogeneity in both mean and plasticity in behaviour needs to be accounted for when predicting species responses to environmental change might have to be considered on a case-by-case basis, and individual plasticities as well as means may be targets of selection and hence evolvable.

## Data Availability

Data and R code to recreate the analysis are available through Figshare [78].
